# *Trypanosoma brucei gambiense*-iELISA: A Promising New Test for the Post-Elimination Monitoring of Human African Trypanosomiasis

**DOI:** 10.1093/cid/ciaa1264

**Published:** 2020-08-28

**Authors:** Manon Geerts, Nick Van Reet, Sander Leyten, Raf Berghmans, Kat S Rock, Theresa H T Coetzer, Lauren E-A Eyssen, Philippe Büscher

**Affiliations:** 1 Department of Biomedical Sciences, Institute of Tropical Medicine, Antwerpen, Belgium; 2 Advanced Practical Diagnostics BVBA, Turnhout, Belgium; 3 Mathematics Institute, University of Warwick, Coventry, United Kingdom; 4 School of Life Sciences, University of KwaZulu-Natal, Pietermaritzburg, South Africa

**Keywords:** *Trypanosoma brucei gambiense*, elimination, ELISA, diagnosis

## Abstract

**Background:**

The World Health Organization targeted *Trypanosoma brucei gambiense* human African trypanosomiasis (*g*HAT) for elimination as a public health problem and for elimination of transmission. To measure *g*HAT elimination success with prevalences close to zero, highly specific diagnostics are necessary. Such a test exists in the form of an antibody-mediated complement lysis test, the trypanolysis test, but biosafety issues and technological requirements prevent its large-scale use. We developed an inhibition ELISA with high specificity and sensitivity that is applicable in regional laboratories in *g*HAT endemic countries.

**Methods:**

The *T. b. gambiense* inhibition ELISA (*g*-iELISA) is based on the principle that binding of monoclonal antibodies to specific epitopes of *T. b. gambiense* surface glycoproteins can be inhibited by circulating antibodies of *g*HAT patients directed against the same epitopes. Using trypanolysis as reference test, the diagnostic accuracy of the *g*-iELISA was evaluated on plasma samples from 739 *g*HAT patients and 619 endemic controls and on dried blood spots prepared with plasma of 95 *g*HAT and 37 endemic controls.

**Results:**

Overall sensitivity and specificity on plasma were, respectively, 98.0% (95% CI 96.7–98.9) and 99.5% (95% CI 98.6–99.9). With dried blood spots, sensitivity was 92.6% (95% CI 85.4–97.0), and specificity was 100% (95% CI 90.5–100.0). The *g*-iELISA is stable for at least 8 months when stored at 2–8°C.

**Conclusion:**

The *g*-iELISA might largely replace trypanolysis for monitoring *g*HAT elimination and for postelimination surveillance. The *g*-iELISA kit is available for evaluation in reference laboratories in endemic countries.

## Teaser

The novel *Trypanosoma brucei gambiense* inhibition ELISA (*g*-iELISA) is a high-throughput diagnostic applicable in regional laboratories for monitoring *gambiense*-HAT elimination. On plasma samples, sensitivity was 98.0% and specificity 99.5%, while on dried blood spots sensitivity was 92.6% and specificity 100%.

Human African trypanosomiasis (HAT) is an infectious disease caused by the protozoan *Trypanosoma brucei* (*T.b.*) [1]. Transmission occurs via infected tsetse (*Glossina sp.*), which confines the disease to sub-Saharan Africa [2]. Two geographically separated sub-species are responsible for the disease in humans: *T.b. gambiense* type I in West and Central Africa and *T.b. rhodesiense* in Eastern Africa [2]. Some atypical human infections are due to another trypanosome taxon, called *T.b. gambiense* type II but are very rare and not considered here [3]. Both *gambiense*-HAT (*g*HAT) and *rhodesiense*-HAT (*r*HAT) are neglected tropical diseases, however, despite this improved control tools for *g*HAT recently appeared, such as rapid diagnostic tests (RDTs) for diagnosis, new drugs including nifurtimox-eflornithine combination therapy (NECT) and fexinidazole for treatment, and tiny targets for vector control. Sustained control activities in most affected countries have reduced annual incidence to a point where elimination of *g*HAT as a public health problem (<2000 reported cases reported annually and 90% reduction of the area at risk reporting ≥1 case/10 000 people/year), seems feasible, and elimination of transmission (EOT, zero human cases of *g*HAT) is now the new target [4–6]. However, as long as tsetse populations subsist, *g*HAT may reappear in foci that are considered eliminated. Re-emergence of the disease may be caused by i) an animal reservoir, although the epidemiological role of animals is still under debate, or ii) a human reservoir in the form of patients that are not picked up by active or passive surveys or asymptomatic carriers that harbor the infection for years or decades without developing the disease [7–9].

Measuring EOT of *g*HAT poses new challenges for diagnosis, especially as this infection often persists at extremely low levels. Likewise, there is a threat of re-emergence or re-invasion from another area with ongoing transmission. In epidemic and endemic situations where the goal is to reduce incidence drastically and rapidly, diagnostics should be highly sensitive, which usually compromises their specificity but results in a high negative predictive value (NPV) following the formula$NPV=\frac{specificity\;\times \;(1\;-\;prevalence)}{\left(1\;-\;sensitivity\right)\;\times \;prevalence\;+\;specificity\;\times \;(1\;-\;prevalence)}.$On the other hand, when prevalence is near zero, it is important to use diagnostics with very high specificity to avoid any false-positive results that may trigger unnecessary alarm and ensuing actions. High specificity results in high positive predictive values (PPV) following the formula$PPV=\frac{sensitivity\;\times \;prevalence}{sensitivity\;\times \;prevalence+\left(1-specificity\right)\;\times \;(1-prevalence)}.$

Parasitological diagnosis of *g*HAT, based on microscopic detection of the parasite in blood, lymph, or cerebrospinal fluid, is highly specific, but moderately sensitive [2]. For this reason, field applicable serological tests have been introduced since the 1970s [10]. They have been instrumental in the control of *g*HAT but are ineffective for *r*HAT. The card agglutination test for trypanosomiasis (CATT), of which several million are used each year, is particularly useful for large-scale screening of populations at risk. The sensitivity and specificity of the test are estimated at 91.2% and 97.4%, respectively [11]. RDTs have been available since 2013. A comparative study on archived specimens from West Africa reported sensitivities of 98.5% for the *gambiense* Sero K-SeT and 99.6% for the SD Bioline HAT; specificities were much lower, 98.6% and 97.1% respectively [12]. With these characteristics, CATT or RDTs cannot be recommended for postelimination monitoring of *gHAT—even* in current screening programs a single RDT or CATT positive test is not alone considered sufficient for administration of treatment—and confirmation by microscopy is required. Diagnostics for an endgame setting must be increasingly specific, otherwise there will overwhelmingly be more false positives than true positives [13, 14]. An alternative could be the variant-specific trypanolysis test (TL) [15]. This antibody-mediated complement lysis test combines high specificity and high analytical sensitivity and is used to confirm the presence of *gambiense*-specific antibodies in CATT or RDT positive individuals but in which the parasite cannot be demonstrated by microscopy or molecular tests [16].

The TL test is recognized by the World Health Organization (WHO) as the reference test for contact with *T.b. gambiense* and, as such, is performed at the WHO Collaborating Centers on HAT (Institute of Tropical Medicine Antwerp, Belgium, and Institut National de Recherche Biomédicale, Democratic Republic of the Congo [DRC]) and in Centre International de Recherche-Développement sur l’Elevage en zone Subhumide, Burkina Faso. The test is applicable in laboratory conditions on serum, plasma, and dried blood spots (DBS) [17]. Its specificity is due to the fact that on intact bloodstream trypomastigotes of *T. brucei*, only the variable antigen type (VAT)-specific epitopes of the variant surface glycoprotein (VSG) coat are accessible to conventional antibodies (IgM and IgG). VAT-specific antibodies in a test specimen will opsonize the trypanosomes that are subsequently lysed by antibody-mediated complement lysis. As most *g*HAT patients have antibodies against the VATs Lille Trypanosome antigen types 1.3 and/or 1.5 (LiTat 1.3, LiTat 1.5), TL is carried out with both variants [15]. The primary disadvantage of TL is that it requires in vivo propagation of highly-virulent *T.b. gambiense* clones, which puts the laboratory personnel under biohazard risk. Secondary disadvantages are the low throughput (400 samples/week) and high cost (5–7 €/test).

We here describe the development of an inhibition ELISA (iELISA) with similar diagnostic accuracy but fewer disadvantages than TL. In the *Trypanosoma brucei gambiense*-iELISA (*g*-iELISA), the binding of monoclonal antibodies (mAbs) to VAT-specific epitopes on the VSGs of *T.b. gambiense* variants LiTat 1.3 and LiTat 1.5, is inhibited by binding of antibodies in the blood of *g*HAT patients directed to the same epitopes.

## MATERIALS AND METHODS

We first established a Target Product Profile (TPP) describing the intended use and test characteristics (Supplementary Material 1). The profile defined for the *g*-iELISA is that of a tool to monitor the progress towards *g*HAT elimination and to assess the presence/absence of *T.b. gambiense* in the human population of a focus where *g*HAT transmission is thought to have stopped. The test must be applicable in national and regional laboratories in sub-Saharan African countries affected by *g*HAT. Serum, plasma, or DBS, collected in local health facilities or through population surveys, are sent to the nearest laboratory able to run the *g*-iELISA. Sensitivity must be at a minimum >90% and optimistically ≥95%, and specificity must be at a minimum ≥99.5% and optimistically 100%. *g*-iELISA stability should be at a minimum 24 months at 4°C and optimistically 24 months at 30°C. The *g*-iELISA will be commercialized as in vitro diagnostic device (IVD) submitted to the European Directive 98/79/EC (IVDD 98/79/EC).

For development of the *g*-iELISA and to define a cut-off value, we used plasma samples collected during a previous study conducted in the DRC [18].

Purified VSGs were produced following Büscher and co-workers with some modifications [19] (Supplementary Material 2). Reactivity of the VSGs was assessed in indirect ELISA by testing 2-fold dilutions, ranging from 4 to 0.125 µg/mL, with both variant-specific mAbs described below.

Mouse mAbs against VSG LiTat 1.3 (clone 7B1D7) and LiTat 1.5 (clone 1A11G10) were generated using standard protocols at Icosagen Cell Factory (Tartumaa, Estonia).

VAT-specific chicken IgY were produced at the University of KwaZulu-Natal. IgY antibodies were affinity purified on VSG LiTat 1.3 and LiTat 1.5 columns and (cross)-reactivity was verified in ELISA (Supplementary Material 3).

The Research Use Only (RUO) prototype *g*-iELISA was developed at Advanced Practical Diagnostics (Supplementary Material 4).

Diagnostic accuracy of this RUO *g*-iELISA (index test) on plasma was assessed on 1358 samples from *g*HAT patients and controls from the WHO Human African Trypanosomiasis Specimen Bank and originating from DRC, Guinea, Chad, and Uganda [20]. Controls were individuals living in endemic areas with negative serology (CATT) and parasitology for *g*HAT, and without previous *g*HAT infection. To assess the diagnostic accuracy of the *g*-iELISA on DBS, samples were prepared with healthy donor blood of which plasma was replaced by plasma of 95 TL-positive *g*HAT patients and 37 endemic controls [18, 21] (Supplementary Material 5).

Trypanolysis was used as reference test. All plasma samples were tested in TL with two VATs of *T.b. gambiense* type I (ie, LiTat 1.3 and LiTat 1.5), according to [15] with a cut-off of 30%.

## RESULTS

### Cut-off Value

**Figure 1.**
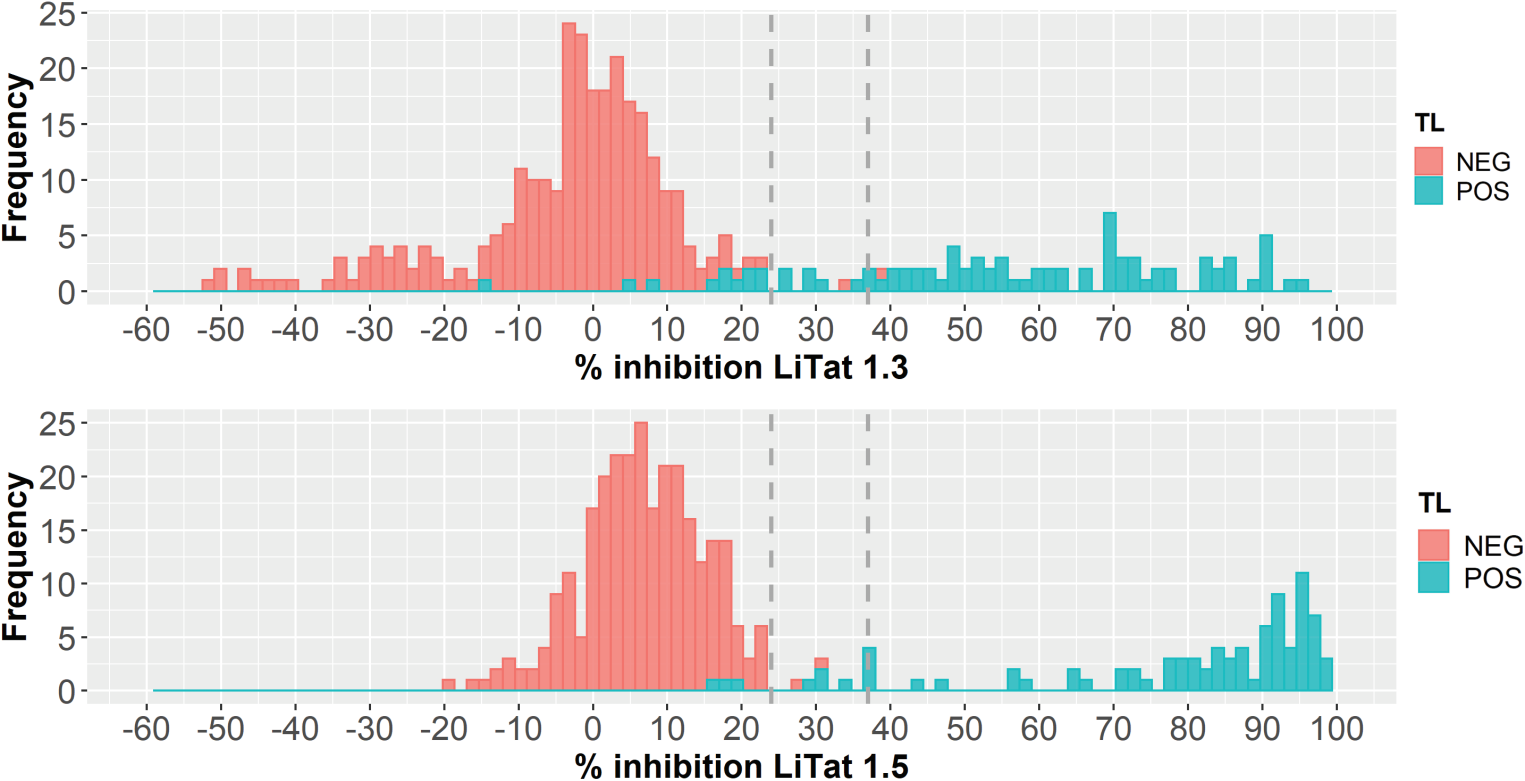
Frequency plots of % inhibition results obtained with 87 TL-positive and 275 TL-negative samples in the *g*-iELISA with LiTat 1.3 and LiTat 1.5 antigen. Dashed lines indicate the cut-off value range with highest Youden index. Abbreviations: TL, trypanolysis test; NEG, negative; POS, positive.

To define a percent inhibition cut-off value, 87 TL-positive and 275 TL-negative samples from DRC were tested. Combining the results obtained with each VSG antigen separately (Figure 1), the sensitivity and specificity were calculated at varying % inhibition cut-off values. The Youden index (sensitivity + specificity—1) was highest between 24% and 37% inhibition cut-off value (0.977 and 0.966, respectively). Percent inhibition values ranged from −52.5% to 95.3% in the test with LiTat 1.3 VSG and from −19.6% to 98.3% in the test with LiTat 1.5 VSG. Despite all attempts to avoid the negative percent inhibition often observed with TL-negative samples and with the LiTat 1.3 VSG, we were not able to overcome this unexpected phenomenon that, on the other hand, did not hinder to score the final result as positive or negative.

### Results on Plasma Samples

All 1358 plasma samples were tested in TL against *T.b. gambiense* LiTat 1.3 and LiTat 1.5. With TL as the reference test and using 35% inhibition as cut-off in the index test, the overall diagnostic accuracy of the *g*-iELISA is 99.5% with a 95% confidence interval (CI) of 99.0% to 99.8 (computed using the exact Clopper-Pearson method), assuming a 0.05% prevalence as reported for active screening [4]. The sensitivity is 98.0%, and the specificity is 99.5% (Table 1). Thus, values for sensitivity and specificity are above the values set in the TPP, ≥95% (optimistic) for sensitivity and (minimum) >99.5% for specificity. This is also the case when stratifying the results per country, except for DRC where observed specificity was slightly lower (99.47%) than the minimum value proposed in the TPP (99.5%).

**Table 1.** Diagnostic Parameters Obtained in *g*-iELISA on Plasma with TL as Reference Test

	TP	FN	TN	FP	Sensitivity %	95% CI	Specificity %	95% CI
All countries	724	15	616	3	98.0	96.7–98.9	99.5	98.6–99.9
DRC	529	13	567	3	97.6	95.9–98.7	99.5	98.5–99.9
Guinea	89	0	30	0	100.0	95.9–100.0	100.0	88.4–100.0
Chad	75	2	19	0	97.4	90.9–99.7	100.0	82.3–100.0
Uganda	31	0	-	-	100.0	88.8–100.0	-	-

Abbreviations: CI, confidence interval; FN, false negative; FP, false positive; TN, true negative; TP, true positive.

### Results on DBS

With TL as reference test and 35% inhibition cut-off in the index test, the diagnostic accuracy of the *g*-iELISA on plasma samples is 100.0% (95% CI 97.2–100.0). The sensitivity is 94.7%, which is somewhat lower than the optimistic value but well above the minimum value (>90.0%) set in the TPP; the specificity is 100% (Table 2). With 20% inhibition cut-off, the diagnostic accuracy on DBS is 100.0% (97.2% to 100.0%) with a sensitivity of 92.6% and a specificity of 100.0% (Table 2). Compared to the sensitivity obtained with the corresponding plasma samples, testing DBS induced a small loss in sensitivity (2.1%) but still above the minimum value set in the TPP. Agreement between results obtained in *g*-iELISA with plasma and DBS is almost perfect (Cohen’s kappa k = 0.897, 95% CI: .73–1.067) [22].

**Table 2.** Diagnostic Parameters Obtained in *g*-iELISA with DBS and Corresponding Plasma Samples

	TP	FN	TN	FP	Sensitivity %	95% CI	Specificity %	95% CI
Plasma	90	5	37	0	94.7	88.1–98.3	100	90.5–100.0
DBS	88	7	37	0	92.6	85.4–97.0	100	90.5–100.0

Abbreviations: CI, confidence interval; FN, false negative; FP, false positive; TN, true negative; TP, true positive.

TL is used as reference test.

### Precision

Repeatability, expressed as coefficient of variation (%CV) of 20 tests on the same sample in the same run, varied between 1.6% with antigen LiTat 1.5 and 5.8% with antigen LiTat 1.3 (Table 3). Reproducibility, expressed as the %CV on 6 samples tested in 4 different runs, varied between 1.7% with LiTat 1.5 and 18.9% with LiTat 1.3 (Table 4).

**Table 3.** Repeatability of Results Obtained in *g*-iELISA by Testing 2 Trypanolysis-Positive Samples 20× in the Same Run

	Antigen LiTat 1.3		Antigen LiTat 1.5	
	Sample 1	Sample 2	Sample 1	Sample 2
Average % inhibition	58.8	78.9	56.0	78.5
Standard deviation	3.4	2.1	2.8	1.2
% coefficient variation	5.8	2.6	5.0	1.6

**Table 4.** Reproducibility of Results Obtained in *g*-iELISA by Testing 6 Trypanolysis-Positive Samples in 4 Different Runs

Antigen LiTat 1.3	Sample 1	Sample 2	Sample 3	Sample 4	Sample 5	Sample 6
Mean % inhibition	60.6	47.9	66.7	43.0	0.2	55.2
Standard deviation	3.5	6.1	5.5	8.1	4.0	7.2
% coefficient variation	5.7	12.8	8.2	18.9	5.7	13.1
Antigen LiTat 1.5	Sample 1	Sample 2	Sample 3	Sample 4	Sample 5	Sample 6
Mean % inhibition	58.2	79.1	80.8	37.5	37.7	70.4
Standard deviation	3.8	2.1	1.4	6.4	5.3	3.2
% coefficient variation	6.5	2.7	1.7	17.2	14.0	4.5

### Stability Testing

**Figure 2.**
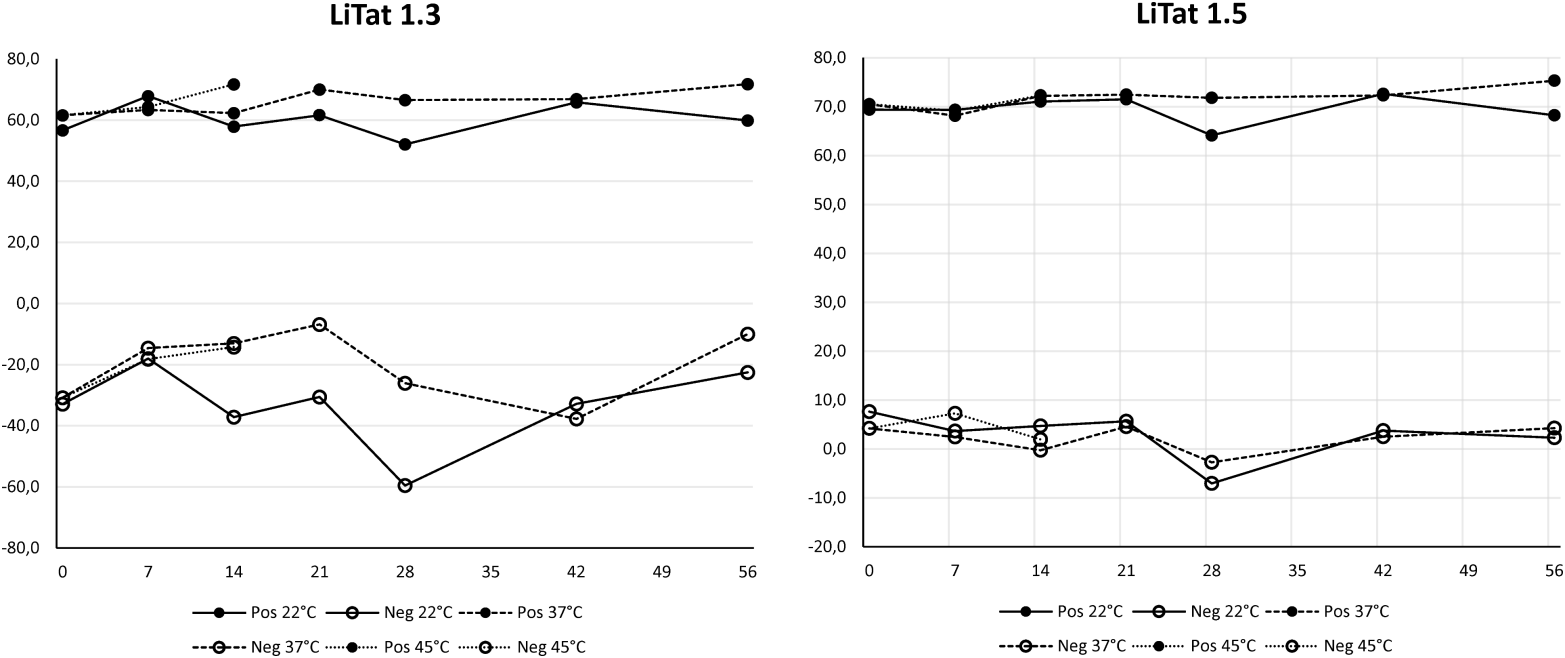
Accelerated stability results. Four trypanolysis-positive and 2 trypanolysis-negative samples were tested with *g*-ELISA kits after 7, 14, 21, 28, 42, and 56 days storage at 22°C and 37°C, and after 7 and 14 days storage at 45°C. Abbreviations: Neg, negative; Pos, positive.

Accelerated stability testing, performed according to ISO 23640, revealed considerable variation in optical density (O.D.) values obtained with samples and controls in the test with LiTat 1.3 antigen, in contrast to LiTat 1.5 antigen. The % inhibition remained stable over time for all storage conditions except for the TL-negative samples tested with LiTat 1.3 antigen (Figure 2).

So far, real-time stability testing of *g*-iELISA kits stored at the storage temperature prescribed in the IFU (2–8°C), showed that the kits remain stable after 8 months storage at that temperature. Stability will further be followed up to 24 months.

## DISCUSSION AND CONCLUSION

This study was undertaken to develop a high-throughput surveillance test for monitoring the EOT of *T.b. gambiense* and to assess its sustained absence or re-emergence in *g*HAT foci in the postelimination phase. Since the test will be deployed in situations where infection rates are very low or zero, it should have a high positive predictive value and therefore a very high specificity to avoid raising the alarm unnecessarily [23]. We opted for an ELISA since this test format is cheap and can be implemented in national and regional laboratories in sub-Saharan Africa, in contrast to TL, which is the WHO recommended reference test for anti-*T.b. gambiense* antibodies in humans, but that is performed in only3 laboratories world-wide. At least 3 times more samples can be tested simultaneously in *g*-iELISA, while for TL the limit is 400 samples per week. Aiming at high specificity of the new test, we designed it as an inhibition ELISA based on the recognition of 2 specific epitopes by two mAbs, each conjugated with horseradish peroxidase; thus i) avoiding reaction of test sample antibodies with other, less specific epitopes present on native VSGs, and ii) obviating the need for host-specific antibody conjugates when testing other host species that may harbor *T.b. gambiense* infections, such as domestic animals. As positive controls, we selected chicken antibodies (IgY) over mammalian antibodies since they can be produced in a less invasive, more cost-effective way, and in much larger amounts. Purified IgYs are stable up to 60°C [24] and remain reactive following storage at 4°C for several years (Coetzer, unpublished observation).

The *g*-iELISA makes use of a combination of native *T.b. gambiense* antigens that are also used in other serodiagnostic tests for *g*HAT, like the RDTs HAT Sero-K-SeT and SD Bioline HAT [25, 26], latex agglutination [27] ,and other ELISAs [28, 29]. None of these tests had a specificity higher than the minimum of 99.5% proposed in the TPP. For example, an ELISA with a mixture of VSGs LiTat 1.3, LiTat 1.5, and LiTat 1.6 was 99.2% (95% CI: 95.7–100) specific with sera from 128 negative controls and 98.7% (95% CI: 93.1–100) sensitive with sera from 78 *g*HAT patients [28], which, at 0.05% prevalence, would yield a PPV of 6.0% (95% CI: .9–30.8%). At the same prevalence, the here described *g*-iELISA on plasma would yield a PPV of 9.2% (95% CI: 3.2–23.8%), which is still far from optimal. Interestingly, maximum PPV was obtained when testing DBS instead of plasma (due to 100% specificity). For large-scale surveillance, the easiest specimen to collect is DBS, which is used in other disease surveillance programs. Active, village-based screening for *g*HAT by means of collecting DBS for remote testing in ELISA or other antibody detection tests has already been proposed a long time ago as an alternative to active screening by mobile teams [23, 30]. Its cost-effectiveness is evaluated in an ongoing study in Côte d’Ivoire, Burkina Faso, Guinea, and DRC (https://www.ditect-hat.eu/). More detailed analysis is required to assess the level of certainty the *g*-iELISA would provide in verifying whether the elimination of transmission goal has been reached using this collection framework. The benefits of other plausible sampling schemes for measurement of the elimination goal could be quantified using state-of-the-art Bayesian statistical frameworks [31], historical data, and mechanistic modelling approaches.

DBS specimen are currently collected in *g*HAT sentinel sites established with the help of the WHO in 15 endemic countries [32]. They are subsequently sent to the few reference laboratories where TL is performed, thus allowing detection of *g*HAT cases in nonendemic foci [33]. Replacing TL by *g*-iELISA opens perspectives to set up a larger network of sentinel sites in the most remote foci of an endemic country linked to national or regional laboratories equipped for ELISA testing. This will cut operational costs and the delay between sampling and test result, which, for obvious reasons, is beneficial to the national or international programs involved in *g*HAT elimination. Operational costs will further be reduced by the lower price of *g*-iELISA (3 €/test) compared to TL (5–7 €/test).

Results obtained with the current version of the *g*-iELISA are promising, in particular since diagnostic accuracy was similar on samples originating from West and Central Africa. However, further investigations may be considered to overcome inherent disadvantages. For instance, accelerated stability testing showed the limited stability of the LiTat 1.3 antigen at 22°C and higher, thus necessitating storage and long-distance transport of the kit between 2°–8°C. Furthermore, the need to cut out 8 × 6 mm diameter discs from each DBS prior to actually testing the eluted fraction, puts a limit on the number of samples that can be tested simultaneously by one lab technician. As an alternative, small blood volumes could be dried in the wells of a microfiltration plate that contains a suitable absorbing filter pad from which the test sample can be eluted via vacuum or centrifugation. Also, replacing the native antigens, which are produced in laboratory rodents, by recombinant antigens or peptides, would be a major achievement in the context of the 3Rs (Replacement, Reduction, and Refinement of animals in research). In previous studies, we developed alternative diagnostic antigens, derived from the VSGs LiTat 1.3 and LiTat 1.5, in the form of synthetic peptides and recombinant antigens produced in *Pichia pastoris* and *Leishmania tarentolae* [34–37]. Unfortunately, none of these antigens reacted with the highly VAT-specific monoclonal antibodies currently used in the *g*-iELISA, thus necessitating the development of new monoclonal antibodies before an inhibition ELISA with these alternative antigens can be constructed.

The present study has a limitation that makes it difficult to compare the sensitivity and specificity of the *g*-iELISA with those reported in other studies on *g*HAT serodiagnostics. Most *g*HAT patients, from which the plasma samples were collected, were screened with CATT/*T.b. gambiense* before undergoing parasitological confirmation. In addition, endemic controls were generally defined as negative in CATT/*T.b. gambiense*. Thus, we observed TL negatives among the *g*HAT patients and TL positives among the controls. However, since the aim was to develop an ELISA with similar characteristics as TL, we used the latter as a reference test and not the positive or negative status in parasitological examination. To assess its clinical accuracy and robustness, the *g*-iELISA should be evaluated under conditions prevailing in reference laboratories in the endemic countries.

In conclusion, the diagnostic sensitivity and specificity of this prototype *g*-iELISA comply with the minimum requirements set in the TPP, in particular when testing with DBS. As such, the test might largely replace the TL for monitoring the *g*HAT elimination progress and for postelimination surveillance. The RUO ELISA kit is available for evaluation in reference laboratories in *g*HAT endemic countries.

## Supplementary Data

Supplementary materials are available at *Clinical Infectious Diseases* online. Consisting of data provided by the authors to benefit the reader, the posted materials are not copyedited and are the sole responsibility of the authors, so questions or comments should be addressed to the corresponding author.

ciaa1264_suppl_Supplementary_File_1

ciaa1264_suppl_Supplementary_File_2

ciaa1264_suppl_Supplementary_File_3

ciaa1264_suppl_Supplementary_File_4

ciaa1264_suppl_Supplementary_File_5

## Notes

***Acknowledgments.*** Plasma samples originate from the WHO HAT Specimen Bank and were provided by Pasteur Institute Paris.

***Disclaimer*.** The funders had no role in the design, decision to publish, or preparation of the manuscript.

***Financial support.*** This work was supported by the Bill & Melinda Gates Foundation [grant numbers OPP1174221 to PB, OPP1177824 to KSR].

***Potential conflicts of interest.*** The authors: No reported conflicts of interest. All authors have submitted the ICMJE Form for Disclosure of Potential Conflicts of Interest.
