# Supplementary material for: Trypanosoma brucei gambiense-iELISA: A Promising New Test for the Post-Elimination Monitoring of Human African Trypanosomiasis
Source: Clin Infect Dis. 2020 Aug 28;73(9):e2477–83. doi: 10.1093/cid/ciaa1264 (PMC8563279; doi:10.1093/cid/ciaa1264)
Supplement: ciaa1264_suppl_Supplementary_File_1 [file ciaa1264_suppl_supplementary_file_1.pdf]

Diagnostic Assay/Instrument - Target Product Profile

Diagnostic Assay/Instrument:
Trypanosoma brucei gambiense-iELISA

Version: V1.0 21-04-2020

Contents

1 Medical Need / Differentiation Strategy/Use Case 4
1.1 Medical Need 5
1.2 Intended Use Case Scenario 6
1.3 Critical Assumptions 7
2 Annotated Product Requirements 8
2.1 TPP Change Management ..... **Error! Bookmark not defined.**

Instructions for Use – Delete/update all blue text when creating document

This template is divided into three sections:

1. *Medical Need/Differentiation Strategy/Use Case*
2. *Product Requirements with Annotations*
    a. *Minimum criteria are the set of performance and use characteristics to achieve the minimally acceptable level of global health impact (based on modeling, uptake, prevention of cases etc.). These criteria provide context for defining clear go/go no decision criteria that can be applied throughout the development process.*
    b. *Optimistic criteria are the set of performance and use characteristics of an optimistic product for which the global health impact should be broader, deeper, quicker, etc.*
    c. *Annotations provide questions to think about and examples for each variable. The teams should use this section as a guide through completion of the TPP, and to capture greater detail and the rationale on the thinking that supports the minimum and optimistic targets captured in the Executive Summary. The expectation is that the teams will be able to speak to this level of detail. (*Variables in the Executive Summary are noted with an asterisk.)*
3. *Change Management section is to capture the changes made to each version of the TPP to enable clear tracking of the evolution of the TPP.*
    a. *Version numbering convention (major/minor as determined by the strategy team)*
    b. *Major version changes should be reflected as V1.0, V2.0, V3.0, etc.*
    c. *Minor version changes should be reflected as V1.1, V1.2, V1.3, etc.*
    d. *Person responsible for maintaining the TPP and making the change should be captured.*

For further details on the TPP Process or Development, please refer to the TPP Business Process.

1 Medical Need / Differentiation Strategy/Use Case

1.1 Medical Need

<<Instructions: Brief description of the medical need and differentiation strategy from existing or projected future interventions i.e. what health problem will it solve and what is its added value beyond other interventions? >>

Human African trypanosomiasis (HAT) caused by *Trypanosoma brucei gambiense* is a vector borne tropical disease and is endemic in West and Central sub-Saharan Africa. Almost half a million *gambiense*-HAT (*g*HAT) cases were diagnosed between 1990 and 2015. Today, this epidemic has been brought under control with <3000 cases in 2016 and the WHO has set the target of eliminating *g*HAT as a public health problem by 2020 and zero transmission by 2030. Given the current low to very low prevalence of the disease in most foci, a highly sensitive and specific test is needed to monitor the process of *g*HAT elimination and, in particular, to assess the presence/absence of *T.b. gambiense* in the human population of a HAT focus where the disease is believed to have been eliminated. Indeed, based on historical evidence, it will be necessary to monitor the presence/absence of *T.b. gambiense* parasites in a so-called "extinct" focus for many years (likely >10) to ensure transmission has ceased. Moreover, with the possibility of an animal reservoir of the parasite, there is also a need to monitor the presence/absence of *T.b. gambiense* in animals, in particular domestic species where potentially overlapping transmission cycles could lead to recrudescence in humans.

For the purpose of post-elimination monitoring, a serodiagnostic test that detects *T.b. gambiense* specific antibodies is the most appropriate since, even in the absence of detectable parasites or clinical symptoms, it can unveil prior or present contact of a host with the parasite. However, with very low to zero expected prevalence, the test should have an extremely high specificity to yield a sufficiently high positive predictive value when used in large-scale population surveillance or sampling. In addition, the test should allow high-throughput testing of dried blood spots (DBS) as this is the most convenient specimen type for large-scale surveys. None of the existing serodiagnostic tests for *T.b. gambiense* fulfils all these requirements - the specificity is either suboptimal, the tests are not appropriate for DBS testing or are not high-throughput.

The *Trypanosoma brucei gambiense*-iELISA (*g*-iELISA) will ideally be 100% specific, will be applicable on DBS and will allow high-throughput testing. Thus, it will become a valuable tool in our toolbox to be deployed for the elimination and post-elimination phases of *g*HAT.

1.2 Intended Use Case Scenario

<<Instructions: Describe the envisioned scenario(s) and the critical assumptions in which this product will be accessed/used. Can it be given to general population or require targeting to certain groups? From what service delivery channels/points – e.g. primary health care, reproductive health facility, retail, campaign, other? Do patients use at the point of care, home, other? How will the test result be integrated in patient management? >>

The *g*-iELISA is intended to be performed in national and regional laboratories in sub-Saharan African countries affected by *g*HAT. Blood on filter paper can be collected by staff in local health facilities or through population or animals surveys by trained teams. This can be collected in even the most remote foci of an endemic country, after which the specimens are sent to the nearest laboratory able to run the *g*-iELISA. This will not only cut operational costs but also the delay between sampling and test result, which, for obvious reasons is beneficial to the national or international programmes involved in *g*HAT elimination. Critical assumptions are 1) a functional country program for collecting and dispatching DBS; 2) functional regional or national laboratories with sufficient infrastructural and personnel capacity to run the *g*-iELISA, 3) a strategy for taking reactive measures in case a positive result is observed in the *g*-iELISA, 4) a long term political and financial engagement to eliminate *g*HAT.

Future mathematical modelling will help to clarify the costs associated with post-elimination surveillance strategies in relation to the information gained, and the certainty that elimination has been achieved in previously endemic foci. Optimal surveillance strategy/sampling schemes have yet to be formulated but modelling and the WHO HAT-e-TAG will provide guidance on suitable measurement for local elimination of transmission.

1.3 Critical Assumptions

<<Instructions: Highlight the critical assumptions used to develop the TPP. The detailed assumptions are captured within the annotations. >>

The work starts from the assumption that it is possible to develop an inhibition ELISA (also called competitive ELISA) using recombinant *T.b. gambiense*-specific variant surface glycoproteins (VSGs) LiTat 1.3 and LiTat 1.5 and their corresponding VAT-specific monoclonal antibodies. The *g*-iELISA will have the following characteristics:

- applicable to humans and other mammals
- applicable on blood collected on filter paper
- based on recombinant antigens and antibodies (lab animal free)
- fully *T.b. gambiense*-specific and not cross-reacting with other human- and animal-infective trypanosomes
- 100% diagnostic specificity, >90% diagnostic sensitivity
- high throughput
- robust and easy to produce
- cheaper than 7 Euro/test (current price of the immune trypanolysis test)

2 Annotated Product Requirements

Variable	Minimum[1]	Optimistic[2]	Annotation[3]
**1. Intended Use**			
1.1 What this test shall detect, what is the target organism and/or molecular component to be detected?	• Host antibodies against *T.b. gambiense*-specific antigens	• Host antibodies against *T.b. gambiense* specific-antigens	• Antigens: Variant Surface Glycoproteins (VSG) LiTat 1.3 and LiTat 1.5 that bear fully *T.b. gambiense* type I-specific epitopes<br>• In the first instance, the antigens will be native; later during development, they can be replaced by recombinants
1.2. Are there variants/genotypes/subtypes to be detected or avoided?	• Target subspecies and type is *T.b. gambiense* type I	• Target subspecies and type is *T.b. gambiense* type I	• Non-*T.b. gambiense* type I infections in humans are very rare and not targeted for elimination<br>• Targeting *T.b. rhodesiense* will cause cross-reactions with the non-human infective *T.b. brucei* and should be avoided<br>• In animals, infections with other trypanosome species should not be detected (*T. congolense, T. vivax, T. evansi, T.b. brucei, T. melophagium, T. theileri, T. simiae* etc.)

[1] Minimal should be considered as a potential go/no go decision point
[2] Optimistic should reflect what is needed to achieve broader, deeper, quicker global health impact; note only additional or different features from minimum)
[3] For all parameters, include here the rationale for why this feature is important and/or for the target value.

Variable	Minimum[1]	Optimistic[2]	Annotation[3]
1.3. What is the intended use population for this test?	• General human population living in active or eliminated *g*HAT foci	• General human and animal populations living in active or eliminated *g*HAT foci	• Considering the possibility of an animal reservoir of *T.b. gambiense* type I, animals, in particular domestic species, should be considered for screening.<br>• Targeting "high risk" strata within the human and animal populations may be optimal
1.4. What is the information to be used for? What is the actionable result? Is this an IVD for diagnosis, screening, prognosis, or monitoring Test? Is this for surveillance, Investigational Use (IUO) or Research Use Only test (RUO)?	• The end product will be an IVD for post-elimination surveillance based on the monitoring of the human population for contact with *T.b. gambiens*e type I<br>• The detection and parasitological confirmation of a seropositive individual should trigger reactive control measures	• The end product will be an IVD for post-elimination surveillance based on the monitoring of the human and animal populations for contact with *T.b. gambiense* type I<br>• The detection and parasitological confirmation of a seropositive case should trigger reactive control measures	• If the *g*-iELISA is 100% specific, a positive case reflects contact with *T.b. gambiense* type I, not necessarily an active infection. However, evidence exists that some human individuals can harbor symptomless infection for several decades. Animals show symptomless infections as well. Both of these groups may transmit back to tsetse creating infectious reservoirs. Moreover, specific antibodies remain detectable for several years in cured HAT patients. Therefore, every single seropositive case should be examined

Variable	Minimum[1]	Optimistic[2]	Annotation[3]
			extensively. If safe drugs are available then treatment of asymptomatic people could be considered, or other controls (such as vector control) could be put in place to avert further human or animal infections in the area.
1.5. What type of specimens will be tested?	• Dried blood spots, serum and plasma	• Dried blood spots, serum and plasma, saliva	• For large-scale surveillance, the easiest specimen to collect is dried blood spot. This type of specimen is currently used for the immune trypanolysis test and is being collected in other disease surveillance programs.<br>• We include saliva as possible specimen although we have evidence that the concentration of specific antibodies is only a fraction of the blood concentration.
1.6. Is this a qualitative or a quantitative test?	• Qualitative	• Qualitative	• Quantification of circulating antibodies is irrelevant for serodiagnosis of *T.b gambiense* type I infection
1.7. What type of platform/technology is used?	• Inhibition Enzyme Linked Immunosorbent Assay	• Inhibition Enzyme Linked Immunosorbent Assay	• This platform allows high-throughput testing and can be

Variable	Minimum[1]	Optimistic[2]	Annotation[3]
			automated. However, automated elution of dried blood spots is challenging and not addressed here.
**2. Individual (Patient) or Population Needs and Performance Characteristics**			
2.1. Clinical sensitivity	• > 90%	• ≥ 95%	• Two factors may have a negative effect on the diagnostic sensitivity of the test: 1) the time needed to build up a detectable level of antibodies against the antigens used in the test; we assume this time is around one month, based on experimental infections in rabbits. 2) The slightly reduced sensitivity of the test, when performed on dried blood spots, compared to plasma or serum, due to incomplete elution of the antibodies from the filter paper.<br>• A general comment on the assessment of the test performance characteristics is the fact that for diagnosis of *T.b. gambiense* type I infection, no single gold standard test

Variable	Minimum[1]	Optimistic[2]	Annotation[3]
			exists. This means that a combined gold standard has to be constructed or that performance characteristics have to be assessed via Bayesian statistics such as latent class analysis.<br>• The higher the sensitivity of the test, the fewer the missed infections. For a post-elimination surveillance program a higher sensitivity will mean the same number of (negative) tests will yield higher certainty of elimination, however the minimum sensitivity required will be determined by the cost, willingness to pay and feasibility of population sampling.
2.2. Clinical specificity	• > 99,5%	• 100%	• Until today, no false positives have been observed in trypanolysis on human specimens. We speculate that we can obtain the same specificity with the *g*-iELISA, at least in humans. In animals, we

Variable	Minimum[1]	Optimistic[2]	Annotation[3]
			cannot yet exclude false positives caused by animal trypanosome infections.<br>• A fully specific test is extremely desirable in both pre- and post-elimination surveillance and could provide a high-certainty early warning that infection is still circulating in the region prior to recrudescence of disease. Even a slightly less specific test will produce many false positive tests given the large-scale nature of surveillance; such positive results would then have to be tested using a fully specific test such as the trypanolysis to confirm or exclude infection. Therefore a less specific test may only be acceptable if costs are low.
2.3. Analytical specificity/cross reactivity	• 100 % analytical specificity in humans	• 100 % analytical specificity in human and animal	• The specificity of the test mainly depends on the specificity of the monoclonal antibodies for their corresponding antigen. To date, no evidence exists that

Variable	Minimum[1]	Optimistic[2]	Annotation[3]
			the variant specific epitope of the VSG antigens are shared with other infectious agents.<br>• One confounding factor that could interfere with analytical specificity is reaction of test sera with mouse antibodies. Solutions to overcome this problem do exist.
2.4. Analytical sensitivity	• Not relevant but can be determined on final prototype, if desired	• Not relevant but can be determined on final prototype, if desired	• An estimation of the minimum concentration of circulating antibodies that can be detected is not possible yet. It will depend on the final set-up of the test<br>• However, since the *g*-iELISA will be a qualitative test, this estimation is not relevant
2.5. Precision & reproducibility	• Overall percent agreement: 99.5% (intra-observer and inter-observer)<br>• Percent positive agreement: 99.95%	• Overall percent agreement: 99.99% (intra-observer and inter-observer)<br>• Percent positive agreement: 99.99%	• Percent positive agreement may become 0% if no HAT cases exist anymore
2.6. Test robustness (interference, cross contamination, validity)	• Possible interference with anti-mouse antibodies in the test specimen	• No interference with other blood constituents	• Risk of interference is reduced by use of HRPO conjugated monoclonal antibodies.

Variable	Minimum[1]	Optimistic[2]	Annotation[3]
	• Cross contamination between test specimens minimized by use of disposable materials during elution of DBS and *g*-iELISA run	• No cross contamination between test specimens<br>• Validity of the *g*-iELISA run tested by inclusion of positive and negative control	• Presence of anti-mouse antibodies in the test specimen can be neutralized by desorption of the DBS eluate with mouse antibody fragments or by humanizing the *gambiense*-specific MAbs.
2.7. What is the risk of an inaccurate result?	• Intrinsic: 10 % false negatives and 1% false positives in human test samples<br>• Inaccurate results can occur when reagents deteriorate.	• Intrinsic: 5% false negatives in human and animal test samples<br>• Inaccurate results can occur when reagents deteriorate.	• see annotation about stability under 2.8
2.8. What are the assay/system features to minimize risk?	• Reagent/test kit stability known	• Reagent/test kit stability known<br>• Positive and negative control specimens included in the test kit	• Stability assessment of the test kit can only be undertaken at a later stage in the test development<br>• The type of control specimens to be included in the test kit has to be decided early in the test development.
**3. Regulatory, Statutory Needs**			
3.1. What type of global and local regulatory approvals and standards (e.g. WHO PQ or other process, Compliance with QSR e.g. FDA QSR; ISO	• CE marking (compliant with the European Directive 98/79/EC (IVDD 98/79/EC)	• CE marking (compliant with the European Directive 98/79/EC (IVDD 98/79/EC)<br>• QMS ISO13485:2016	• The *g*-iELISA is an *in vitro* diagnostic medical device (IVD) submitted to the European Directive 98/79/EC (IVDD

Variable	Minimum[1]	Optimistic[2]	Annotation[3]
13485:2003) are needed before commercialization in different countries?	• QMS ISO13485:2016		98/79/EC) if it will be placed on the European market or if it will be used within Europe. Having the CE mark facilitates registration – or might even be a prerequisite – in an increasing number of African countries (McNerney and Peeling, CID 2015:61 (Suppl 3) S135-S140, DOI: 10.1093/cid/civ553).<br>• Compliance with IVD Directive includes the implementation of a quality management system, which will be ISO 13485:2016. This includes application of GMP
3.2. What type of promotional, educational, marketing & sales materials are allowed?	• All documentation having product information compatible with the content of the Technical File is allowed. The Technical File is established according to the IVD Directive and ISO 13485:2016 when the product is CE marked.	• All documentation having product information compatible with the content of the Technical File is allowed. The Technical File is established according to the IVD Directive and ISO 13485:2016 when the product is CE marked.<br>• Peer reviewed publications	• Product information (flyer, IFU) will also be available on the website of the manufacturer.

Variable	Minimum[1]	Optimistic[2]	Annotation[3]
	• Peer reviewed publications		
**4. Healthcare System Needs**			
**4.1 Environment Description**			
4.1.1Where the test is to be performed? At what HC level? What are the environmental conditions?	• Regional and national laboratory<br>• Storage of tests at 4-8°C<br>• Operating of test at 18-25°C and 80% humidity	• Regional, national and provincial laboratory<br>• Storage of tests at 18-37°C<br>• Operating of test at 18-37°C and 90% humidity	• All laboratories that fulfill the minimum requirements regarding electricity, source of pure water, cold chain, trained personnel, are eligible for performing the test. However, in most countries, these labs are only present in the capital cities.<br>• The temperature of the tests in transit is an important consideration. In the minimum scenario a cold-chain will be vital and could create some challenges in transport of tests to some endemic regions.<br>• The product is designed for professional use only.<br>• Transport conditions will be determined before placing the product on the market, based on real kit transport conditions and on simulated problematic conditions.

Variable	Minimum[1]	Optimistic[2]	Annotation[3]
**4.2. Instrument & Device Characteristics**			
4.2.1. Instrumentation physical dimensions (HxWxL); modularity; weight; and level of automation	• ELISA reader: 13x45x37 cm<br>• ELISA washer: 17x50x40 cm<br>• Plate incubator: 45x40x40 cm<br>• Fridge: 188x61x70 cm<br>• Desktop: 48x45x48 cm<br>• All instruments are installed separately. ELISA reader and washer are semi-automated.<br>• Manual filter paper elution.	• ELISA reader: 13x45x37 cm<br>• ELISA washer: 17x50x40 cm<br>• Desktop: 48x45x48 cm<br>• All instruments are installed separately. ELISA reader and washer are semi-automated.	• Fully integrated ELISA systems exist but are not recommended for installation in sites where maintenance and repair facilities are not available locally<br>• Small lab equipment like precision balances, magnetic stirrer, micropipettes etc. are not considered since should be available in clinical laboratories
4.2.2. Instrumentation power and water requirements	• Power and pure water required	• Power and pure water required	• We presume that a water distillation apparatus or other source of pure water is available locally
4.2.3. Instrument reliability & maintenance requirements	• Maintenance and spare parts required	• Maintenance and spare parts required	• This is a critical issue in the laboratories in endemic countries
4.2.4. Workflow requirements. What type of throughput is needed? How fast the result is needed?	• Workflow can be separated in specimen collection, shipment to the laboratory, specimen	• Workflow can be separated in specimen collection, shipment to the laboratory, specimen preparation and actual ELISA assay	• In trypanolysis, about 400 specimens can be tested per week<br>• If, in ELISA, specimens are tested in duplicate, 720

Variable	Minimum[1]	Optimistic[2]	Annotation[3]
	preparation and actual ELISA assay<br>• ELISA plates are coated just before the assay or coated and stored frozen<br>• Medium throughput<br>• One month between collection and test result	• ELISA plates are precoated and stored at longer term<br>• High throughput<br>• One week between collection and test result	specimens can be tested per week (6 days)<br>• In a post-elimination setting the timescale is less important, and even the minimum (1 month) between collection and result should be acceptable to find and treat the case prior to severe disease given the typical progression of *gambiense*-HAT. Patients in late stage disease would likely self-present to medical facilities and be diagnosed and treated using the existing passive surveillance system.
4.2.5. Waste management & biosafety requirements	• Standard biosafety precautions for handling potentially infectious materials	• Standard biosafety precautions for handling potentially infectious materials	• ELISA assay is associated with considerable non-degradable waste (plastics, reagents)
**4.3. Information & Communication Technology**			
4.3.1. User interface and data input requirements	• Quantitative optical density readout with ELISA reader<br>• Electronic database	• Quantitative optical density readout with ELISA reader<br>• Electronic database	• Further simplification of the assay without ELISA apparatus requirement may be considered
4.3.2. Data output, access, security, storage and	• To be determined	• To be determined	• To be worked out

Variable	Minimum[1]	Optimistic[2]	Annotation[3]
communication (connectivity) requirements			
4.3.3. How are the results transmitted?	• Email, local access to central server, phone	• SMS, phone	• To be further worked out
**4.4 Reagent and control handling**			
4.4.1. How shall the reagents/cartridges/controls be stored? How shall they be packaged?	• Test pack size = 30 tests per individual plate<br>• Plate to prepare locally<br>• Reagent dilutions to prepare locally<br>• Kit stability: 24 months at 4-8°C	• Test pack size = 30 tests per individual sealed, plate<br>• Sealed plate ready to use<br>• Reagents pre-diluted<br>• Test kit stability: 24 months at 30°C	• Number of specimens that can be tested per plate depends on whether test is run in duplicate or not<br>• The tests consists of at least two independent tests, one with LiTat 1.3 and one with LiTat 1.5; a third test without antigen is included in the prototype but may prove to be redundant in the final test format. In that case, the number of samples tested per plate will be 40.
**4.5. Sample Handling**			
4.5.1. What type of specimens and assays are to be run in the same facility? How are the specimens tracked and received?	• Routine clinical laboratory specimens.<br>• According to routine clinical lab conditions.	• Routine clinical laboratory specimens.<br>• According to routine clinical lab conditions.	• Risk of cross-contamination is low<br>• The test will require dedicated equipment<br>• Ideally an L2 lab, however this could be challenging in many endemic countries

Variable	Minimum[1]	Optimistic[2]	Annotation[3]
4.5.2. Sample type(s) and volumes	• Dried blood spots, serum or plasma<br>• 100 µl	• Dried blood spots, serum or plasma, saliva<br>• 30 µl	• Typically dried blood spots contain 30 µl of blood of which only one fourth is eluted for indirect ELISA; we expect to test lower dilutions without risk of non-specific reactions
4.5.3. Sample collection & transport requirements	• Venous blood collected on site, plasma prepared and shipped frozen	• Blood from finger prick or saliva, spotted on filter paper and shipped on silica gel after drying at ambient temperature	• Special collecting devices exist that can be considered but are rather expensive<br>• Training for collection will be required
4.5.4. Sample preparation requirements	• Extraction from filter paper, serum or plasma dilution	• Extraction from filter paper, serum, plasma or saliva dilution	• Extracted specimen from filter paper can be frozen if needed
**4.6. Distribution, Service & Support, Training**			
4.6.1. Who will run the test? How he/she will be trained and supported?	• Laboratory technician<br>• Specific training in specimen preparation and ELISA, including digital data calculation	• Laboratory technician<br>• Specific training in specimen preparation and ELISA, including digital data calculation	• Dedicated data input/output applications will have to be programmed<br>• A positive test should trigger follow-up of the case and the population in the area (such as a reactive screen). The collection team or health facility as well as the national sleeping sickness control program must be alerted

Variable	Minimum[1]	Optimistic[2]	Annotation[3]
4.6.2. What type of Quality Control System is needed to monitor test/site performance on ongoing bases? What other support is needed?	• Negative and positive control for test performance assessment<br>• Proficiency panel for site performance	• Negative and positive control for test performance assessment<br>• Proficiency panel for site performance	• Establishing a proficiency panel for HAT diagnosis has never been done before. It may not contain specimen from human origin for biosafety reasons<br>• Stability is an issue<br>• Replicate tests should be performed in the case of uncertain results
4.6.3. Instrument & test supply reliability	•	•	• To be worked out
4.6.4. Service & support response time	•	•	• To be worked out
**5. Commercial and Sustainability Needs**			
5.1. In what countries will be launched? Is controlling the disease a priority for the government of the countries we intend to supply? Are their timelines in alignment with ours? Is advocacy needed?	• Countries endemic for *g*HAT in West and Central-Africa that reported cases in the last decade and with functional reference laboratories	• All countries endemic for *g*HAT in West and Central-Africa that reported cases in the last decade	• See London Declaration<br>• Advocacy certainly needed<br>• Priority countries include those which are very close or may already have locally eliminated *gambiense*-HAT. These are currently: Benin, Togo, The Gambia, Senegal, Burkina Faso, Côte d'Ivoire, Nigeria etc….
5.2. What are the funding agencies that would support it? Are there any IP global access	• WHO, Belgian Cooperation, BMGF	• WHO, Belgian Cooperation, BMGF	• Other funding agencies may join

Variable	Minimum[1]	Optimistic[2]	Annotation[3]
issues? Who would negotiate them?			
5.3. What are commercial channels?	• Directly to end-user.	• Directly to end-user.	• Manufacturer has a distributer in Nigeria.
5.4. What does the total end-user price per test (reagents & consumables; ex-works) need to be? What is the maximum cost of the instrumentation (per module as applicable)?	• US$3.00/test<br>• Full ELISA chain and fridge costs about 20,000 $	• US$1.00/test<br>• ELISA chain without fridge and incubator costs about 15,000 $	• Test price depends on whether specimens are tested in duplicate or not<br>• Cost for instruments is based on European prices<br>• Other costs include shipment of equipment<br>• The acceptable cost of the test will interplay with the test qualities (sensitivity and specificity) and the price countries and the global community are willing to pay for high certainty of elimination
