# Supplementary material for: Trypanosoma brucei gambiense-iELISA: A Promising New Test for the Post-Elimination Monitoring of Human African Trypanosomiasis
Source: Clin Infect Dis. 2020 Aug 28;73(9):e2477–83. doi: 10.1093/cid/ciaa1264 (PMC8563279; doi:10.1093/cid/ciaa1264)
Supplement: ciaa1264_suppl_Supplementary_File_2 [file ciaa1264_suppl_supplementary_file_2.pdf]

Preparation of experimental dried blood spots

To assess the diagnostic accuracy of the *g*-iELISA on dried blood spots (DBS), experimental DBS were prepared as follows.

Venous blood was taken on heparin from a trypanolysis-negative O+ donor and divided into 500 µl volumes in microfuge tubes. After centrifugation for 10 min at 17,000*g* and ambient temperature, 250 µl of the plasma in each tube were replaced by plasma of a *g*HAT patient or an endemic control. With each blood sample, 6 volumes of 70 µl were spotted on Whatman 4 filter paper, dried overnight at ambient temperature and stored in plastic bags with silica gel to keep the filters dry until use. As test samples for the *g*-iELISA, 8 discs of 6 mm diameter were punched out from each filter and eluted overnight in 400 µl of iELISA sample diluent. In total, we prepared DBS with plasma from 95 *g*HAT patients from Nord Equateur Province in DRC, all positive in trypanolysis, and from 37 endemic controls from Kwilu Province in DRC, all negative in trypanolysis.
