# Supplementary material for: Trypanosoma brucei gambiense-iELISA: A Promising New Test for the Post-Elimination Monitoring of Human African Trypanosomiasis
Source: Clin Infect Dis. 2020 Aug 28;73(9):e2477–83. doi: 10.1093/cid/ciaa1264 (PMC8563279; doi:10.1093/cid/ciaa1264)
Supplement: ciaa1264_suppl_Supplementary_File_3 [file ciaa1264_suppl_supplementary_file_3.pdf]

Preparation of purified variant surface glycoproteins

Rats were inoculated intraperitoneally with cloned *T.b. gambiense* VAT LiTat 1.3 or LiTat 1.5 [1]. After 3 days, trypanosomes were harvested from the blood by DEAE-cellulose chromatography [2]. After 3 washings with cold phosphate buffered saline supplemented with glucose (0.05 mol/L phosphate, 0.036 mol/L NaCl, 0.0833 mol/L glucose, pH 8.0), the sediment from the last centrifugation step was suspended in 6 volumes of 10 mM phosphate buffer pH 8.0 causing hypotonic lysis. Lysed trypanosome suspension was centrifuged (120 minutes, 42,000*g*, 4°C). The supernatant was dialysed against 10 mM phosphate buffer pH 8.0 and fractionated on a DEAE cellulose column equilibrated with the same buffer. The first elution fraction containing the VSG was collected. After measuring the protein concentration by UV spectrophotometry, the VSG was stored at -20°C until use. The purity of the VSG preparations was assessed by reducing SDS-PAGE and Coomassie blue staining (**see figure**).

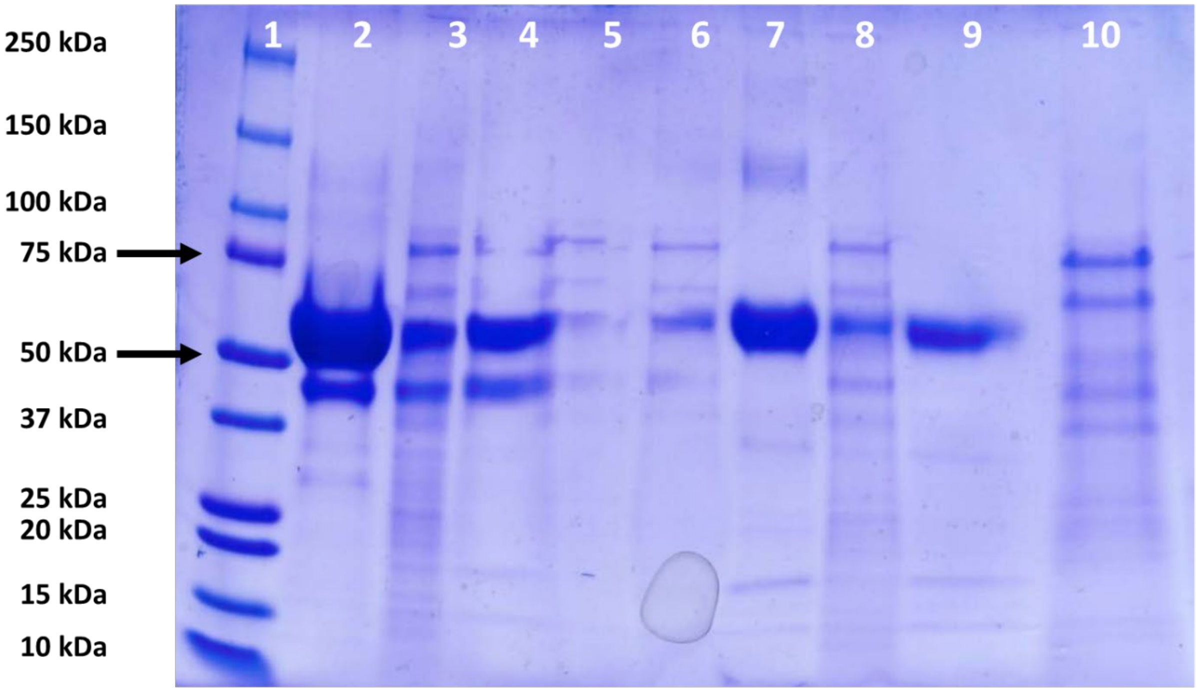

Coomassie blue stained reducing SDS-PAGE of protein fractions collected during the purification of VSG LiTat 1.5 (lanes 2-6) and VSG LiTat 1.3 (lanes 7-10). Lane 1: Molecular mass marker, lane 2: purified VSG LiTat 1.5 (positive control), lane 3: total water soluble trypanosome lysate, lane 4: fraction with purified VSG, lane 5 and lane 6: non-VSG fraction, lane 7: purified VSG LiTat 1.3 (positive control), lane 8: total water soluble trypanosome lysate, lane 9: fraction with purified VSG, lane 10: non-VSG fraction.

1. Magnus E, Vervoort T, Van Meirvenne N. Serological cross-reactions among trypanosome variable antigen isotypes of the subgenus *Trypanozoon*. Annales de la Société Belge de Médecine Tropicale **1982**; 62: 25-39.
2. Lanham SM, Godfrey DG. Isolation of salivarian trypanosomes from man and other mammals using DEAE-cellulose. Experimental Parasitology **1970**; 28: 521-34.
