# Supplementary material for: Trypanosoma brucei gambiense-iELISA: A Promising New Test for the Post-Elimination Monitoring of Human African Trypanosomiasis
Source: Clin Infect Dis. 2020 Aug 28;73(9):e2477–83. doi: 10.1093/cid/ciaa1264 (PMC8563279; doi:10.1093/cid/ciaa1264)
Supplement: ciaa1264_suppl_Supplementary_File_4 [file ciaa1264_suppl_supplementary_file_4.pdf]

Preparation of VAT-specific chicken IgY

VAT-specific chicken IgY were produced at the University of KwaZulu-Natal. Approval for the protocols involving the housing and immunisation of chickens was obtained from the University of KwaZulu-Natal Animal Research Ethics Committee (Approval number 053/15/Animal) that adheres to SANS 10386:2008 South African National Standard for the Care and Use of Animals for Scientific Purposes. Laying hens were immunised with 50 μg purified VSG LiTat 1.3 or VSG LiTat 1.5 per immunisation. After three boosts, IgY was purified from their egg yolks using polyethylene glycol 6000 precipitation following standard protocols [1]. Antibody production and reactivity with the VSG used for immunisation was monitored by ELISA following coating microtitre plates with 1 μg/ml purified VSG LiTat 1.3 and VSG LiTat 1.5. Purified LiTat1.3 and LiTat1.5 (1.5 mg per 0.5 mL resin) was each coupled to AminoLink™ Plus coupling resin (Thermo Scientific, Rockford, IL USA) following the manufacturer's instructions. The isolated anti-LiTat1.3 and anti-LiTat1.5 IgY was each passed over the corresponding affinity matrix, bound antibodies eluted with low pH buffer (100 mM glycine-HCl, 0.02% (w/v) NaN_3, pH 2.8) and collected into neutralisation buffer (1 M NaH_2PO_4, 0.02% (w/v) NaN_3, pH 8.5). Reactivity and cross-reactivity of the affinity purified antibodies with purified VSG LiTat 1.3 and LiTat 1.5 were verified in ELISA (**see figures**).

1. Goldring JPD, Coetzer THT. Isolation of chicken immunoglobulins (IgY) from egg yolk. Biochemistry and Molecular Biology Education **2003**; 31: 185-7.

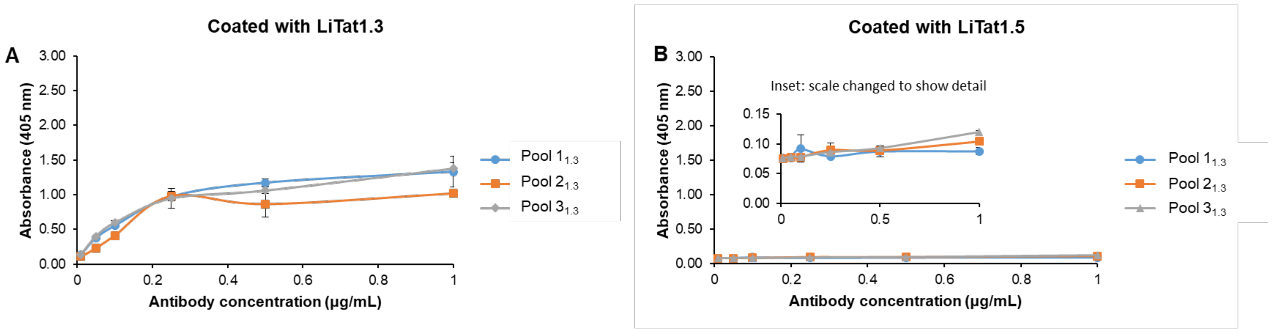

ELISA of affinity purified chicken anti-LiTat-1.3 (Pools $1_{1.3}$-$3_{1.3}$) IgY reactivity with (A) LiTat-1.3 and (B) cross-reactivity with the LiTat-1.5 antigen. ELISA plates were coated with LiTat1.3 **(A)** or LiTat1.5 **(B)** (1 µg/mL in PBS; 100 µL) and probed with of dilutions of affinity purified anti-LiTat1.3 IgY Pools 1-3 prepared in PBS-0.5% (w/v) BSA. Rabbit anti-chicken IgY-HRPO conjugate [1:2500] and chromogen-substrate solution (0.05% (w/v) ABTS, 0.0015% (v/v) H_2O_2 in 100 mM citrate-phosphate buffer, pH 5.0) were used to develop the reaction for 10 min in the dark. The absorbance readings at 405 nm represent the average of triplicate experiments. (PBS, phosphate buffered saline, pH 7.4)

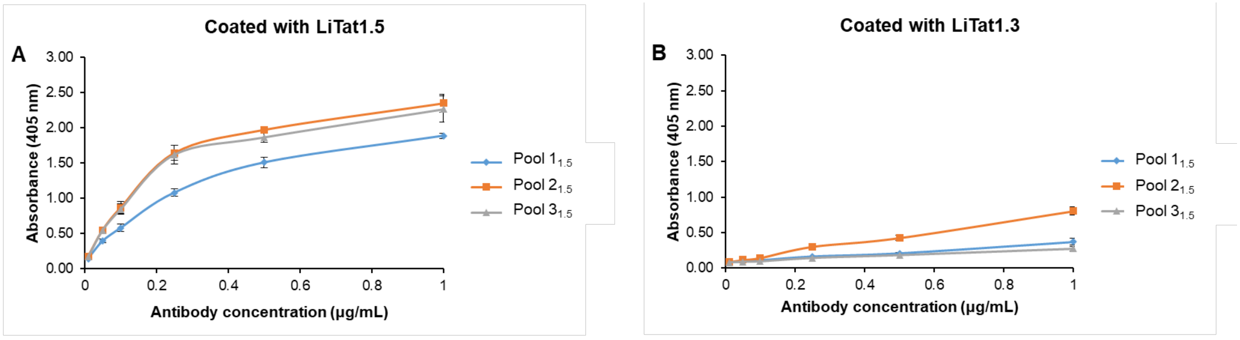

ELISA of affinity purified chicken anti-LiTat-1.5 (Pools $1_{1.5}$-$3_{1.5}$) IgY reactivity with (A) LiTat-1.5 and (B) cross-reactivity with the LiTat-1.3 antigen. ELISA plates were coated with LiTat1.5 **(A)** or LiTat1.3 **(B)** (1 µg/mL in PBS; 100 µL) and probed with of dilutions of affinity purified anti-LiTat1.5 IgY Pools 1-3 prepared in PBS-0.5% (w/v) BSA. Rabbit anti-chicken IgY-HRPO conjugate [1:2500] and chromogen-substrate solution (0.05% (w/v) ABTS, 0.0015% (v/v) H_2O_2 in citrate-phosphate buffer, pH 5.0) were used to develop the reaction for 10 min in the dark. The absorbance readings at 405 nm represent the average of triplicate experiments.
