# Supplementary material for: Trypanosoma brucei gambiense-iELISA: A Promising New Test for the Post-Elimination Monitoring of Human African Trypanosomiasis
Source: Clin Infect Dis. 2020 Aug 28;73(9):e2477–83. doi: 10.1093/cid/ciaa1264 (PMC8563279; doi:10.1093/cid/ciaa1264)
Supplement: ciaa1264_suppl_Supplementary_File_5 [file ciaa1264_suppl_supplementary_file_5.pdf]

Trypanosoma brucei gambiense iELISA

RUO

apDia bvba, Raadsherenstraat 3, 2300 Turnhout, Belgium

Trypanosoma brucei gambiense iELISA
REF 650601 RUO

The apDia *T.b.gambiense* iELISA is an inhibition enzyme linked immunosorbent assay intended for the qualitative measurement of antibodies directed to *T.b. gambiense* LiTat 1.3 and LitTat 1.5 VSG, in serum, plasma or dried blood spots.

PRINCIPLE OF THE *TRYPANOSOMA BRUCEI GAMBIENSE* iELISA

Microtiter strips coated with a mixture of native Variant Surface Glycoproteins (VSG) LiTat 1.3 and LiTat 1.5 from *T.b. gambiense* trypanosomes, are incubated with controls and diluted test samples. During this incubation step, antibodies directed to VSGs LiTat 1.3 and LiTat 1.5 bind specifically to the antigens on the solid phase forming antigen-antibody complexes. After removal of the unbound antibodies by a washing procedure, specific horseradish peroxidase (HRP)-conjugated monoclonal antibodies (clone 7B1D7 directed to LiTat 1.3 VSG and clone 1A11G10, directed to LiTat 1.5 VSG), are added to the wells.
The conjugated monoclonal antibodies can bind directly to the free antigens on the solid phase but cannot bind to the antigen-antibody complexes.
After removal of the unbound conjugates by a washing procedure, the strips are incubated with a chromogenic solution containing tetramethylbenzidine (TMB) and hydrogen peroxide, a blue colour develops if the HRP conjugate is bound. The signal output inversely correlates with the amount of antibody in the samples: the higher the specific antibody concentration, the weaker the output signal. The enzymatic reaction is stopped by the addition of 0.5M H_2SO_4 (changing the TMB colour to yellow) and the absorbance values at 450 nm are determined.

REAGENTS

Component	Name + Symbol
1 coated microtiter plate (12 x 8-well strips)<br>Microtiter strips coated with a mixture of native VSGs LiTat 1.3 and LiTat 1.5.	Precoated strips<br>MTP
1 vial, 0.4 ml, ready-to-use<br>Positive control for *T.b. gambiense* LiTat 1.3 contains IgY from immunised chickens purified by affinity chromatography. Contains 0,09% NaN_3 and a blue dye	Positive control LiTat 1.3<br>POS CTL LiTat 1.3
1 vial, 0.4 ml, ready-to-use<br>Positive control for *T.b. gambiense* LiTat 1.5 contains IgY from immunised chickens purified by affinity chromatography. Contains 0,09% NaN_3 and a red dye	Positive control LiTat 1.5<br>POS CTL LiTat 1.5
1 vial, 1 ml, ready-to-use<br>Negative control for *T.b. gambiense* contains sample dilution buffer with 0.09% NaN_3 and an orange dye.	Negative control<br>NEG CTL
1 bottle, 30 ml, ready-to-use<br>Sample dilution buffer<br>Contains 0.09% NaN_3 and an orange dye.	Sample diluent<br>DIL
1 bottle, 10 ml, ready-to-use<br>Peroxidase conjugated monoclonal anti-LiTat 1.3 clone 7B1D7 antibodies.<br>Contains ProClin 300 as antimicrobial agents and a blue dye.	Conjugate 1<br>CONJ 1 LiTat 1.3
1 bottle, 10 ml, ready-to-use<br>Peroxidase conjugated monoclonal anti-LiTat 1.5 clone 1A11G10 antibodies.<br>Contains ProClin 300 antimicrobial agents and a red dye.	Conjugate 2<br>CONJ 2 LiTat 1.5
1 bottle, 50 ml, 20x concentrated<br>Contains detergent in phosphate buffered solution and ProClin 300 as antimicrobial agents.	Wash solution<br>WASH 20x
1 vial, 20 ml, ready to use<br>Contains a solution of substrate (H_2O_2) and chromogen (tetramethylbenzidine).	Chromogen solution<br>CHROM
1 bottle, 20 ml, ready to use<br>Contains 0.5 M H_2SO_4	Stop solution<br>STOP
3 plate covers	-

1. MATERIALS REQUIRED BUT NOT SUPPLIED

1. Precision micropipettes and standard laboratory pipettes.
2. Clean standard laboratory volumetric glassware.
3. Disposable plastic tubes and tube racks for the dilution of the samples.
4. Vortex or similar mixing tools to mix dilutions of samples.
5. A microtiter plate reader capable of measuring absorbance at 450 nm with reference filter at 600-650 nm.

2. WARNINGS AND PRECAUTIONS FOR USER

1. For Research Use Only (RUO).
2. Wear disposable gloves and protective clothing when performing a test run.
3. Do not mix reagents or coated microtiter strips from kits with different lot numbers.
4. Some kit components contain sodium azide as a preservative. In order to prevent the formation of potentially explosive metal azides in laboratory plumbing, flush drains thoroughly after disposal of these solutions.
5. Although it might be advised to run controls and samples in duplicate, reliable results are equally obtained without replicates.
6. Chromogen solution contains the hazardous ingredient N-Methyl-2 pyrrolidone at a concentration > 0.3 %. It is classified as a reproductive toxicant Category 1B.
   Following hazard statements are applicable: H360D may damage the unborn child.
   Following precautionary statements are applicable:
   P280: wear protective gloves/protective clothing/eye protection/face protection.
   P308+P313: if exposed or concerned, get medical advice/attention
7. Inform the manufacturer in case broken vials/bottles or improperly sealed/closed components are found.

3. STORAGE CONDITIONS

2°C 8°C

1. Store the microtiter strips in their original package with the desiccant until all the strips have been used.
2. Opened components should be stored at 2-8°C until next use and can be maintained for 1 month. The microtiter strips must not be re-used.
3. Never use any kit components beyond the expiry date.

4. SPECIMEN COLLECTION AND PREPARATION

Serum and plasma samples may be used in this assay. Remove serum from clot as soon as possible to avoid haemolysis. Transfer the serum to a clean storage tube. Specimens may be stored at 2-8 °C for 3-4 days, or they can be stored frozen for a longer period of time. Avoid repeated freezing and thawing. Samples must be diluted in sample diluent.

5. ASSAY PROCEDURE

5.1 General remarks

1. Use a disposable tip for each sample transfer to avoid cross-contamination.
2. All reagents must be allowed to come to room temperature before use. All reagents must be mixed gently without foaming.
3. Once the assay has been started, all steps should be completed without interruption and respecting the recommended incubation times.
4. The use of an ELISA washer is recommended, however depending on the apparatus it may be necessary to adapt the washing procedure for obtaining optimal results.

5.2 Reconstitution of reagents

Washing solution: dilute 25 ml of concentrated Wash solution (WASH 20x) to 475 ml with distilled water. Reconstituted solution can be stored at least 1 month at 2-8 °C.
At higher temperatures, the concentrated washing solution may appear cloudy without affecting its performance. Upon dilution, the solution will be clear.

5.3 Assay procedure

Microtiter strips, coated with a mixture of native LiTat 1.3 and LiTat 1.5 VSG, are incubated with controls and diluted samples. Strips 1-6 are intended for detecting antibodies against LiTat 1.3 and strips 7-12 are intended for detecting antibodies against LiTat 1.5.
Specific components (controls and conjugates) are colour coded: blue colour for LiTat 1.3 specific reagents and red colour for LiTat 1.5 specific reagents.

1. Dilute each sample 1:20 in the Sample diluent (DIL), e.g. 25 µl sample + 475 µl DIL
2. Pipette 150 µl of the Negative control (NEG CTL), Positive controls (POS CTL LiTat 1.3 and POS CTL LiTat 1.5) in duplicate, and diluted samples (S1-S44) into the corresponding wells according to the template below.
3. Cover the microtiter strips and incubate them for 60 ± 2 min at ambient temperature (18-25°C).
4. Empty the wells entirely by aspiration. Fill the wells completely with 350 µl of 1:20 diluted Washing solution (WASH), avoiding overflow of buffer from one well to another. Empty the wells by aspiration and repeat the washing procedure two more times for a total of three washes. Finally, aspirate the content of the wells and remove any residual liquid by gently tapping the inverted wells on clean absorbent paper. Incomplete washing will adversely affect the test outcome.
5. Add 150 µl of Conjugate solution 1 (CONJ 1 LiTat 1.3) to all the wells of the strips 1-6 and add 150 µl of Conjugate solution 2 (CONJ 2 LiTat 1.5) to all the wells of the strips 7-12. Cover the microtiter strips and incubate them for 30 ± 2 min at ambient temperature.
6. Repeat the washing procedure as described in 4.
7. Add 150 µl of Chromogen solution (CHROM) to each well.
8. Incubate for 10 ± 1 min at ambient temperature. Avoid light exposure during this step e.g. by placing the microtiter strips in the dark.
9. Add 150 µl of Stop solution (STOP) to each well.
10. Determine the absorbance of each well at 450 nm with reference filter 600-650 nm within 30 min following the addition of Stop solution.

	LiTat 1.3 Measurements						LiTat 1.5 Measurements					
	1	2	3	4	5	6	7	8	9	10	11	12
A	NEG CTL		POS CTL LiTat 1.3		S1	S2	NEG CTL		POS CTL LiTat 1.5		S1	S2
B	S3	S4	S5	S6	S7	S8	S3	S4	S5	S6	S7	S8
C	S9	S10	S11	S12	S13	S14	S9	S10	S11	S12	S13	S14
D	S15	S16	S17	S18	S19	S20	S15	S16	S17	S18	S19	S20
E	S21	S22	S23	S24	S25	S26	S21	S22	S23	S24	S25	S26
F	S27	S28	S29	S30	S31	S32	S27	S28	S29	S30	S31	S32
G	S33	S34	S35	S36	S37	S38	S33	S34	S35	S36	S37	S38
H	S39	S40	S41	S42	S43	S44	S39	S40	S41	S42	S43	S44

6. RESULTS

6.1 Validation of the run

The results for the controls should be within the acceptance criteria before any sample result can be interpreted:
The following specifications must be met for each run to be valid:
OD value for the Negative control: 1.000 < OD < 3.100 (or according to reader capacity).
Percent inhibition for POS CTL LiTat 1.3 and LiTat 1.5: > 30%
If one of the specifications is not met, the test run should be repeated.

6.2 Calculation

Consider the OD-value of the NEG CTL as the well where 100% binding of the conjugated monoclonal antibodies occurs. Calculate for each test sample the percent inhibition of monoclonal antibody binding by using the following formula:

% inhibition = 100 – (OD sample / OD Negative control *100)

6.3 Cut-off value

Depending on the obtained % inhibition, the sample will be considered:
Positive, if % inhibition of LiTat 1.3 **and/or** LiTat 1.5 is ≥ 30%
Negative, if % inhibition of LiTat 1.3 **and** LiTat 1.5 is < 30%

7. PERFORMANCE CHARACTERISTICS

Example of typical optical density (OD) values and % inhibition of Positive control LiTat 1.3 and LiTat 1.5:

**CONTROLS**	**OD**
NEG CTL	2.535
POS CTL LiTat 1.3	0.641
% Inhibition: 74.7 %	

**CONTROLS**	**OD**
NEG CTL	1.820
POS CTL LiTat 1.5	0.794
% Inhibition: 56.4 %	

Precision

Intra-assay variation (n=20; 1 run)

LiTat 1.3	Sample 1	Sample 2
Mean (% inhibition)	58.8	78.9
SD	3.4	2.1
**% CV**	**5.8**	**2.6**

LiTat 1.5	Sample 1	Sample 2
Mean (% inhibition)	56.0	78.,5
SD	2.8	1.2
**% CV**	**5.0**	**1.6**

Inter-assay variation (n=24; 4 runs)

LiTat 1.3	Sample 1	Sample 2	Sample 3	Sample 4	Sample 5	Sample 6
Mean (% inhibition)	60.6	47.9	66.7	43.0	70.2	55.2
SD	3.5	6.1	5.5	8.1	4.0	7.2
**% CV**	**5.7**	**12.8**	**8.2**	**18.9**	**5.7**	**13.1**

LiTat 1.5	Sample 1	Sample 2	Sample 3	Sample 4	Sample 5	Sample 6
Mean (% inhibition)	58.2	79.1	80.8	37.5	37.7	70.4
SD	3.8	2.1	1.4	6.4	5.3	3.2
**% CV**	**6.5**	**2.7**	**1.7**	**17.2**	**14.0**	**4.5**

8. TROUBLESHOOTING

When the OD value of the Negative control exceeds the ELISA reader capacity, repeat the run with shorter incubation time of Chromogen solution.
Example: reduce the incubation time for Chromogen solution from 10 to 6 minutes.

TBG04-20/RUO
